# High Inferior Vena Cava Diameter with High Left Ventricular End Systolic Diameter as a Risk Factor for Major Adverse Cardiovascular Events, Cardiovascular and Overall Mortality among Chronic Hemodialysis Patients

**DOI:** 10.3390/jcm11185485

**Published:** 2022-09-19

**Authors:** Chung-Kuan Wu, Noi Yar, Zih-Kai Kao, Ming-Tsang Chuang, Tzu-Hao Chang

**Affiliations:** 1 Division of Nephrology, Department of Internal Medicine, Shin-Kong Wu Ho-Su Memorial Hospital, Taipei 111, Taiwan; 2 School of Medicine, Fu-Jen Catholic University, New Taipei 242, Taiwan; 3 College of Management, School of Health Care Administration, Taipei Medical University, Taipei 110, Taiwan; 4 Institute of Biophotonics, National Yang Ming Chiao Tung University, Taipei 112, Taiwan; 5 Clinical Data Center, Office of Data Science, Taipei Medical University, Taipei 110, Taiwan; 6 Graduate Institute of Biomedical Informatics, Taipei Medical University, Taipei 110, Taiwan

**Keywords:** IVCD, LVESD, mortality, MACE, hemodialysis

## Abstract

Background: Little is known about the association of inferior vena cava diameter (IVCD) and left ventricular end-systolic diameter (LVESD) with mortality in patients undergoing hemodialysis (HD). Methods: The single medical center observational cohort study enrolled 241 adult chronic HD patients from 1 October 2018 to 31 December 2018. Echocardiography results of IVCD and LVESD prior to dialysis were retrieved and patients were divided into high IVCD and low IVCD groups. Patients who received HD via a tunneled cuffed catheter were excluded. Study outcomes included all-cause mortality, cardiovascular mortality, and major adverse cardiovascular events (MACE). Subgroup analyses of HD patients with high and low LVESD were also performed. Results: The incidence of all-cause mortality, cardiovascular mortality, and MACE were higher in chronic HD patients with high IVCD (*p* < 0.01). High IVCD patients had significantly greater all-cause mortality, cardiovascular mortality, and MACE (log-rank test; *p* < 0.05). High IVCD patients are also associated with an increased risk of all-cause mortality and MACE relative to low IVCD patients (aHRs, 2.88 and 3.42; 95% CIs, 1.06–7.86 and 1.73–6.77, respectively; all *p* < 0.05). In the subgroup analysis of patients with high or low LVESD, the high IVCD remained a significant risk factor for all-cause mortality and MACE, and the HR is especially high in the high LVESD group. Conclusions: Dilated IVCD is a risk factor for all-cause mortality and MACE in chronic HD patients. In addition, these patients with high LVESD also have a significantly higher HR of all-cause mortality and MACE.

## 1. Introduction

Patients with chronic kidney disease (CKD) are at higher mortality risk compared with the general population [1]. Cardiovascular disease represents the leading cause of mortality in these patients, especially those with hemodialysis (HD) or peritoneal dialysis [2,3]. In the population, a positive sodium balance and the expansion of extracellular volume usually exist [4]. Higher amounts of fluid gain are associated with morbid conditions, such as anasarca, pulmonary congestion, hypertension, and worsening heart failure, which are associated with a poor survival rate and increased cardiovascular death [5,6].

The compensation of increased cardiac workload caused by increase preload (volume overload) in HD patients leads to the development of left ventricular hypertrophy (LVH) and subsequent dilatation and dysfunction [7]. Fluid build-up in end-stage kidney disease (ESKD) patients requires maintenance dialysis treatment [8]. The management of extra-fluid volume and hemodynamic instability in HD patients is an essential component of high quality dialysis. Central venous pressure (CVP) is used in clinical practice to assess the volume status and cardiac preload, which can be helpful in the diagnosis and management of the fluid status [9,10,11,12]. The inferior vena cava diameter (IVCD) and collapsibility are clinical outcomes that have been proposed to monitor intravascular volume and right atrial pressure or central venous pressure in dialysis patients [13,14,15]. The IVC is a highly compliant vessel that changes its diameter and cross-sectional area in parallel with changes in blood volume and CVP. Accuracy in IVC measurement has clinical implications in the diagnosis and management of CVD because it affects the estimation of right-sided cardiac pressure [16]. IVCD has been shown to be correlated with the clinical evolution of acute decompensated heart failure (ADHF), evidenced by improvements in diameter and collapsibility after diuretic treatment [17,18,19,20].

Studies have shown that hemodialysis can reduce myocardial blood flow [21,22,23]. In some patients, the fall in myocardial blood flow was severe enough to result in left ventricular systolic dysfunction (LVSD). Reduced ejection fraction (EF) is a sign of apparent left ventricle (LV) dysfunction and LVESD is negatively associated with the recovery of the left ventricular EF and the progression of LV dysfunction [24]. In addition, heart failure or reduced left ventricular ejection fraction (LVEF) in patients with ESKD are associated with a poor prognosis and mortality rates [25,26,27]. All-cause mortality after 2.8 years of follow-up were 52% for patients with left-sided heart disease and 32% for patients without [28]. Additionally, the 1-mm left ventricular end-systolic diameter (LVESD) increment was associated with a 7% increase in overall mortality and a 13% increase in cardiac mortality. Thus, a LVESD > 40 mm was associated with an approximate doubling of the overall mortality risk [29]. However, studies assessing the interaction between IVCD, LVESD, and cardiovascular events are lacking. The objective of the current study was to evaluate the association between IVCD and LVESD with all-cause mortality in patients undergoing HD.

## 2. Materials and Methods

### 2.1. Study Population

**Figure 1:**
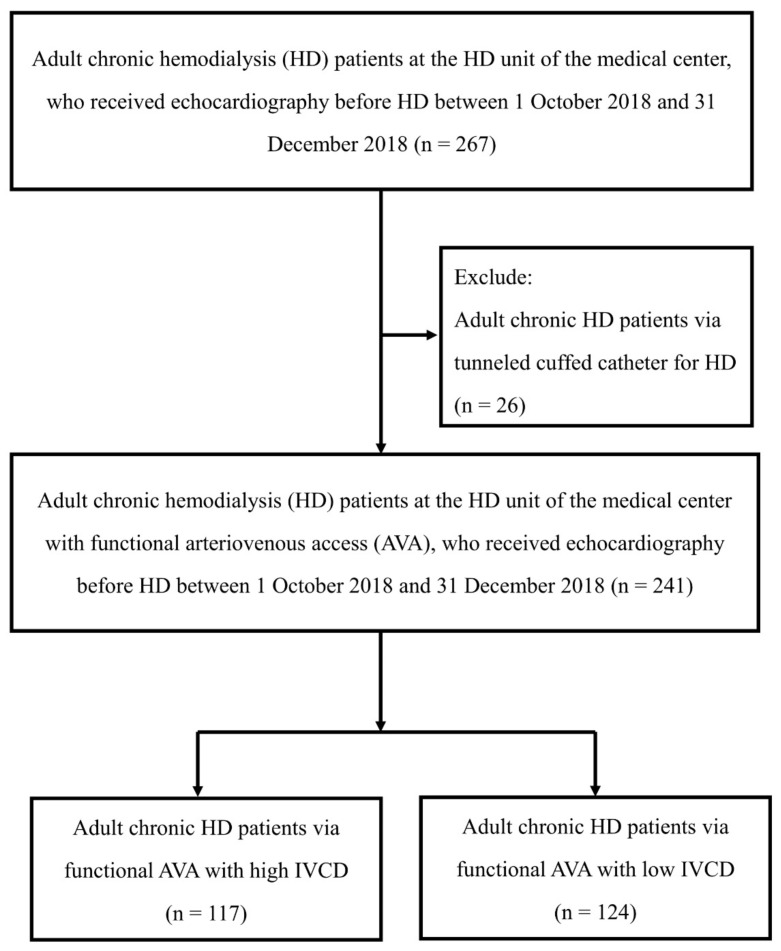
Flow of patient selection for the study cohort. HD, hemodialysis; AVA, arteriovenous access; IVCD, inferior vena cava diameter.

We conducted a single-center observational cohort study. The patient selection process for the cohort study is illustrated in Figure 1. Adult patients who underwent chronic HD treatment via functional AVA at the HD unit of the medical center between 1 October 2018 and 31 December 2018 were enrolled in the study. Chronic HD patients who had echocardiography prior to hemodialysis during the period were included. In addition, patients who received HD via a tunneled cuffed catheter were excluded. A total of 241 adult chronic HD patients who had functional AVA and an echocardiography examination were enrolled in the study. The observation period was from the date of the echocardiography measurement until the end of 2020 or the time of death, whichever occurred first. These patients were divided into high IVCD and low IVCD groups according to a cut-off point of 1.5 cm. These patients with high or low IVCD were sub-grouped into high LVESD and low LVESD according to a cut-off point of 31 mm. The study was approved by the Institutional Review Board of the medical center (No 20211205R and No 20220713R).

### 2.2. History Collection and Laboratory Data

Demographic and baseline clinical data of chronic HD patients were recorded at the time of study recruitment. Data included age, gender, height, weight, comorbid disease history, serum total protein, serum albumin, aspartate aminotransferase (AST), alkaline-P, total bilirubin, serum cholesterol, serum triglyceride, fasting sugar, hemoglobin, serum platelet, iron profile, serum aluminum, serum uric acid, sodium, potassium, ionized calcium, phosphate levels, HD efficiency (Kt/V), and intact parathyroid hormone (iPTH). Blood samples were collected after a fasting period of at least 8 h before each HD session. Kt/V was determined according to the Gotch and Sargent formula [30]. Blood pressure and hypotension during HD were recorded in the first HD session after the measurement of IVCD and LVESD. Meanwhile, conductivity, treatment time, and frequency of HD, interdialytic weight gain, and ultrafiltration in this HD session were also collected. Medications included antihypertensive, antidiabetic, antiplatelet, and anticoagulant drugs.

### 2.3. Measurement of IVCD and LVESD

A standardized transthoracic echocardiography was performed by experienced cardiologists. The echocardiographic examination of IVC was performed the day before HD. A transducer was placed in the subxiphoidal region by the cardiologist to obtain long and short axis views of the IVC. IVCD was measured during expiration and maximal inspiration to avoid Valsalva-like effects via an M-mode echocardiogram before the P-wave on the continuous ECG and to avoid interference with the a-wave and v-wave on the venous pressure curve. The two-dimensional guided M-mode echocardiographic study of the LV was performed in the parasternal long-axis view at the tips of the mitral valve leaflets. LVESD were recorded as the mean values measured in five consecutive cardiac cycles, in accordance with the recommendations of the American Society of Echocardiography [31]. By using the ROC curve, the IVCD and LVESD optimal cut-off points to classify the subjects with outcomes and without outcomes were found to be 1.5 cm and 31 mm, respectively. Identifying the cut-off point minimizes the difference between sensitivity and specificity values.

### 2.4. Study Outcomes

The study outcomes for chronic HD patients in low IVCD and high IVCD groups included all-cause mortality, cardiovascular mortality, and MACE. In addition to IVCD, outcomes in chronic HD patients with high and low LVESD were evaluated.

### 2.5. Statistical Analysis

The patients’ baseline characteristics are summarized as percentages for categorical data and mean (SD) ± standard deviations for continuous data. chi-squared tests or Fisher’s exact tests, which were used for observed values of less than 5, were used to compare categorical variables, and *t*-tests or Kruskal–Wallis tests were used to compare continuous variables, respectively. The patient outcomes between high IVCD and low IVCD groups were compared using a chi-squared test. The survival experiences of all-cause mortality, cardiovascular mortality, and MACE mortality during the follow up were calculated using the Kaplan–Meier method and compared between the high IVCD and low IVCD groups with a log-rank test. A univariate Cox regression analysis was used to estimate the relative risk (crude [HR]) of all-cause mortality, cardiovascular mortality, and MACE mortality in high IVCD and low IVCD patients. Confounders, including age, sex, and all significant variables listed in Table 1, were adjusted in the multivariate Cox proportional hazard models to estimate the adjusted hazard ratios (aHRs). A subgroup analysis was performed to determine the effects of IVCD on the risk of all-cause, cardiovascular, and MACE mortality and a Cox regression analysis was used to calculate the hazard ratio. A forest plot was used to show the results of the subgroup analysis. All statistical analyses were performed using SAS version 9.4 (SAS Institute, Cary, NC, USA). Two-sided *p*-values < 0.05 were considered statistically significant.

## 3. Results

### 3.1. Baseline Characteristics of the Study Population

A total of 241 adult chronic HD patients who had functional AVA and an echocardiography examination before HD and between 1 October and 31 December 2018 were enrolled in the study. Table 1 includes baseline characteristics between high IVCD (*n* = 117) and low IVCD (*n* = 124) groups of chronic HD patients with functional AVA. High IVCD patients (age 63 ± 12 years) were younger than low IVCD patients (68 ± 12 years). Most of the high IVCD patients were male (64% vs. 36% female) and among low IVCD patients, 45% were male and 55% were female, respectively. The mean height for high IVCD patients was 163 ± 9 cm, while low IVCD patients had a mean height of 160 ± 8 cm (all *p* < 0.05). The high IVCD group was followed-up for 26.0 ± 4.8 months while low IVCD group was followed up for 24.5 ± 6.7 months

The prevalence of coronary artery disease was greater among the high IVCD patients (*p* = 0.037). Other comorbid diseases, except for cerebrovascular accidents, COPD, and malignancy, were more prevalent among high IVCD patients, but they were not statistically significant between groups. Chronic HD patients with high IVCD compared with low IVCD had increased total protein (*p* = 0.048), alkaline-P (*p* = 0.035), and PTH (*p* = 0.018); whereas, low IVCD patients had higher cholesterol (*p* = 0.018) and platelet count (*p* = 0.035). The mean total protein was 6.9 ± 0.6 g/dL for high IVCD patients, and it was 6.8 ± 0.5 g/dL for low IVCD patients. High IVCD patients had an alkaline-P of 78.2 ± 36.4 IU/L, while the value for low IVCD patients was 66.6 ± 24.7 IU/L. The PTH level among high IVCD patients was 327.6 ± 306.9 pg/mL and was 245 ± 250.9 pg/mL for low IVCD patients. The serum cholesterol level and platelet count were 151.7 ± 32.6 mg/dL and 179.4 ± 54.0 × 1000/μL, respectively, for high IVCD patients; low IVCD patients had a serum cholesterol level of 163.4 ± 39.0 mg/dL and a platelet count of 201.8 ± 58.1 × 1000/μL. High IVCD patients had significantly higher systolic blood pressure, diastolic blood pressure, and mean arterial pressure than patients with low IVCD. The results were 147.89 ± 23.96 mmHg, 70.28 ± 13.99 mmHg, and 96.13 ± 15.34 mmHg in high IVCD patients and 140.52 ± 25.56 mmHg, 66.30 ± 14.53 mmHg, and 91.02 ± 16.45 mmHg in low IVCD patients, respectively. Of the high IVCD patients, 103 (88%) patients received HD treatment for 4 h, three (2.6%) patients received HD treatment between 3.5 and 4 h, and 11 (9.4%) patients received HD treatment from 3.0 to 3.5 h. Among low IVCD patients, 85 (68.5%) patients received HD treatment for 4 h, three (2.4%) patients received HD treatment between 3.5 and 4 h, and 36 (29%) patients received HD treatment from 3.0 to 3.5 h. The ratio of HD treatment frequency revealed statistical significance between two groups (*p* < 0.001). The prevalence of insulin and analogues and antiplatelet medications was significantly higher in high IVCD patients compared to low IVCD patients. No significant difference of antihypertensive medications, oral antidiabetic medications, and anticoagulants were noted between two groups.

The echocardiographic features between the high IVCD and low IVCD groups have been shown in Table 2. There was no significant difference in the aortic root and relative wall thickness between the two groups. Patients with high IVCD had a significantly greater interventricular septum, left atrium diameter, left ventricular end-diastolic diameter, left ventricular end-systolic diameter, left ventricular posterior wall, left ventricular mass, and left ventricular mass index than those with low IVCD (all *p* < 0.001).

### 3.2. Association between Mortality and IVCD of HD Patients with Functional AVA

The comparison of all-cause mortality, cardiovascular mortality, and MACE between chronic HD patients with low IVCD and high IVCD groups is shown in Table 3. All-cause mortality, cardiovascular mortality, and MACE were all significantly greater in high IVCD patients compared with low IVCD patients. All-cause mortality, cardiovascular mortality, and MACE were 22%, 17%, and 43% for high IVCD patients. Mortality in low IVCD patients was 8.9% for all-cause, 6.5% for cardiovascular, and 17% for MACE.

**Figure 2:**
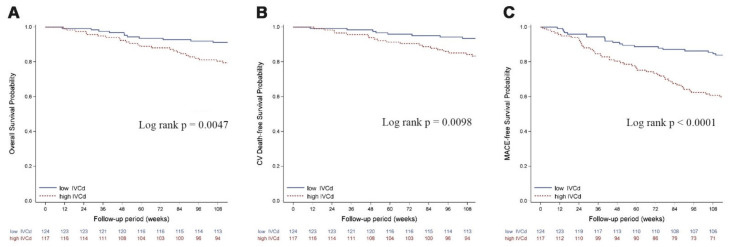
Survival curve for (**A**) all-cause mortality, (**B**) CV mortality, and (**C**) MACE-free events between chronic HD patients in high- and low-IVCD groups.

The Kaplan–Meier survival curve shows that all-cause mortality, cardiovascular mortality, and MACE were higher among the high IVCD group (Figure 2). Log-rank *p* values were all less than 0.05 for all-cause mortality, cardiovascular mortality, and MACE-free events survival. The survival probability curves were significantly lower for high IVCD patients compared with low IVCD patients.

### 3.3. Impact of IVCD on Outcome of HD Patients with Functional AVA

HRs (with 95% CI) from the Cox regression analysis for all-cause mortality, cardiovascular mortality, and MACE events are shown in Table 4. High IVCD patients had at least two times the hazard compared to low IVCD patients. Relative to low IVCD patients, the HRs of high IVCD patients were 2.66 (95% CI 1.31–5.38) for all-cause mortality, 2.81 (95% CI 1.24–6.49) for cardiovascular mortality, and 2.95 (95% CI 1.77–4.92) for MACE-event mortality. After an adjustment for age, sex, coronary artery disease, total protein, alkaline-P, cholesterol, PTH, MAP, dialysis treatment time, insulin and analogues, and antiplatelets, adjusted HRs for high IVCD patients were 2.88 (95% CI 1.06–7.89) and 3.42 (95% CI 1.73–6.77) for all-cause mortality and MACE-event mortality, respectively. *P* values were <0.05 for all-cause mortality and <0.001 for MACE-events.

### 3.4. Subgroup Analysis

**Figure 3:**
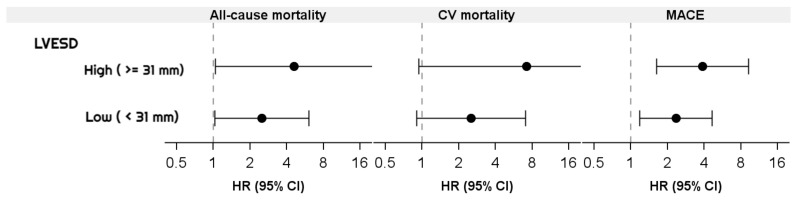
Forrest plot for all-cause mortality, CV mortality, and MACE among IVCD between high and low LVESD groups. CV, cardiovascular; MACE, major adverse cardiovascular events; IVCD, inferior vena cava diameter; LVESD, left ventricular end-systolic diameter; HR, hazard ratio; CI, confidence interval.

The subgroup Cox regression analysis for chronic HD patients with IVCD between LVESD with all-cause mortality, cardiovascular mortality, and MACE is shown in Table 5. The high LVESD with high IVCD group had higher all-cause mortality (HR-4.60, 95% CI: 1.04–20.4) and MACE (HR-3.88, 95% CI: 1.63–9.28) than the high LVESD with low IVCD group. Among the low LVESD patients, all-cause mortality and MACE were significantly higher in high IVCD patients than low IVCD patients (HR-2.50 [95% CI: 1.03-6.09] and HR-2.34, [95% CI: 1.18–4.66], respectively). There was a significant interaction effect between IVCD and LVESD in cardiovascular mortality and MACE (both *p* < 0.05). The high LVESD (≥31 mm) group had significantly higher mortality than the low LVESD group in all-cause mortality and MACE events (Figure 3).

## 4. Discussion

In this observational cohort study, chronic HD patients with high IVCD were younger, taller, and male, with higher proportions of coronary artery disease, insulin and analogues, antiplatelets, total protein, alkaline-P, PTH, higher blood pressure, longer dialysis treatment time, and lower cholesterol and platelet count. Incidence of all-cause mortality, cardiovascular mortality, and MACE were greater in chronic HD patients with high IVCD. During the follow-up, high IVCD patients had poor all-cause mortality, cardiovascular mortality, and MACE-free event survival. Patients in the high IVCD group were also associated with a significantly increased risk of all-cause mortality and MACE. A subgroup analysis of patients with LVESD found that the high IVCD group had increased all-cause mortality and MACE events compared with the low IVCD group.

Complicated with intradialytic hypotension and significant hemodynamic effects caused by intermittent HD treatments, HD patients are particularly prone to impaired vasoregulation, microcirculation, peripheral arterial compliance, and demand myocardial ischemia [32]. Fluid overload caused by fluid retention and chronic inflammation in chronic HD patients could lead to pulmonary congestion, acute pulmonary edema, hypertension, left ventricular hypertrophy, and heart failure [33], which are the risk factors for cardiovascular morbidity and mortality. Therefore, fluid overload may also be an important risk factor for cardiovascular morbidity and all-cause mortality in HD patients [34,35]. In addition, fluid overload is also associated with an increased risk of cardiovascular mortality in HD patients [6,36]. The management of the fluid status is a significant clinical challenge in patients who undergo HD treatment. IVCD can be used to estimate fluid status and intravascular volume in ESKD patients undergoing HD and has been associated with fluid status in adult HD patients [37,38,39]. Dilated IVC is commonly seen in patients with dialysis-associated hypertension [13]. Reduction in dry weight can improve blood pressure control in hypertensive hemodialysis patients, and the adjustment of dry weight based on interdialytic IVCD measurement has been found to improve volume overload and cardiac function in HD patients [40]. IVC dilatation >21 mm has been reported to be associated with all-cause mortality in heart failure patients with impaired renal function, and the prognosis worsened as eGFR declined [41]. Nath et al. also found that a dilated inferior vena cava without collapse with inspiration is associated with an increased risk of mortality [42]. These findings are in agreement with the results of the current study, in which high IVCD is associated with increased cardiovascular mortality and all-cause mortality among chronic HD patients. Published literature focusing on the association between IVCD and all-cause and cardiovascular mortality in HD patients, but not IVCD and MACE, is available. This study noted that high IVCD is significantly related to increased MACE in HD patients.

Chronic fluid overload in ESKD patients significantly elevates cardiac workload, and then induces LVH and left ventricular dilatation over time. The measurement of cardiac dimension is essential in the evaluation of ventricular function. The most commonly used parameters to assess left ventricular systolic function are ejection fraction (EF) and fractional shortening (FS), and LVESD is used for the calculation of FS [43]. Low EF among ESKD patients is associated with a greater risk of cardiovascular death and mortality [25]. LVESD ≥ 40 mm has been found to be independently associated with increased all-cause mortality and cardiovascular mortality in patients with mitral regurgitation [29]. However, there are limited studies regarding the association of LVESD with cardiovascular mortality and all-cause mortality in chronic HD patients. The current study demonstrated that high LVESD has a synergic effect on MACE and all-cause mortality risk among chronic HD patients with IVCD.

Fluid assessment in dialysis is carried out by several methods, including clinical assessment, IVC measurement, bioimpedance spectroscopy, lung ultrasound, and biomarkers, like atrial natriuretic peptide levels [37]. Bioimpedance is the most widely used methods for the hydration status measurement. In pediatric patients, IVCD was found to be correlated with resistance measured by bioimpedance [44]. IVCD measurements by echocardiography reflect the volume status in HD patients [13] and, in combination with other methods, such as clinical parameters and lung ultrasound, etc., it can be useful for determining and adjusting the ultrafiltration volume in fluid removal. However, the reliability of the sonographic IVCD assessment may be reduced due to technical errors, inter-operator variability, and variability with the timing of post dialytic evaluation. Accordingly, in the future, IVCD measurement by echocardiography can be used as a support tool to guide fluid management in a clinical setting if the aforementioned factors can be under control.

Our study has some limitations that need to be considered. Due to retrospective nature of this cohort study, there are some unmeasured confounders, and the statistical quality is lower than for a prospective study. Our study did not include inflammatory markers and precise nutritional markers, which may have influenced the fluid status, cardiac contractility, and remodeling. In addition, our study did not analyze the collapsibility index as it was not included in our echocardiography report. The data are from a single center in Taiwan with a Han Chinese study population, and thus the results may not be generalizable. Baseline echocardiography was measured at study onset, however no longitudinal changes in IVCD or LVESD levels over time were recorded. Echocardiography was scheduled on the date before hemodialysis, but patients may have different interdialytic intervals (long interdialytic intervals or short interdialytic intervals), and interdialytic weight gain (IDWG) may have an influence on IVCD and LVESD.

## 5. Conclusions

Dilated IVCD (≥1.5 cm) is associated with an increased risk of all-cause mortality and MACE-event, and poor survival in chronic HD patients. In addition, patients with high IVCD and high LVESD have significantly greater all-cause mortality and MACE. IVCD measurements may be useful for decreasing the mortality risk in chronic HD patients, either in fluid control with patient education, or for adjustments in dry weight when deemed necessary. LVESD can also be a useful parameter in the evaluation of cardiac function, and for the necessary intervention to lower the risk of MACE in these patients. Further studies with longitudinal measurements of IVCD or LVESD to clarify the independent role of baseline IVCD or LVESD are needed for further validation.

## Figures and Tables

**Table 1:** Baseline characteristics of chronic hemodialysis (HD) patients with functional arteriovenous access (AVA) between high- and low-IVCD groups. IVCD, inferior vena cava diameter.

Variables	High IVCD (*n* = 117)	Low IVCD (*n* = 124)	*p* Value
Age (years)	63.3 ± 12.1	68.0 ± 12.4	0.005 *
Male (%)	75.0 (64.1)	56.0 (45.2)	0.003 †
Female (%)	42.0 (35.9)	68.0 (54.8)	
Height	163.4 ± 8.9	160.0 ± 8.2	0.004 *
Weight	61.6 ± 14.3	58.1 ± 12.9	0.090 *
Comorbid condition			
Diabetes mellitus (%)	57.0 (48.7)	47.0 (37.9)	0.090 †
Hypertension (%)	92.0 (78.6)	97.0 (78.2)	0.940 †
Hyperlipidemia (%)	67.0 (57.3)	57.0 (46.0)	0.080 †
Coronary artery disease (%)	55.0 (47.0)	42.0 (33.9)	0.037 †
Cerebrovascular accident (%)	2.0 (1.7)	3.0 (2.4)	1.000 ‡
PAD (%)	31.0 (26.5)	26.0 (21.0)	0.310 †
Heart failure (%)	28.0 (23.9)	20.0 (16.1)	0.130 †
COPD (%)	8.0 (6.8)	17.0 (13.7)	0.080 †
Malignancy (%)	12.0 (10.3)	15.0 (12.1)	0.650 †
Lab data			
Total protein (g/dL)	6.9 ± 0.6	6.8 ± 0.5	0.048 *
Albumin (g/dL)	3.9 ± 0.3	3.9 ± 0.4	0.350 *
AST (IU/L)	16.4 ± 5.9	16.2 ± 5.2	0.700 *
Alkaline-P (IU/L)	78.2 ± 36.4	66.6 ± 24.7	0.035 *
Total bilirubin (mg/dL)	0.6 ± 0.3	0.5 ± 0.1	0.110 *
Cholesterol (mg/dL)	151.7 ± 32.6	163.4 ± 39.0	0.018 *
Triglyceride (mg/dL)	128.0 ± 84.9	146.2 ± 122.1	0.140 *
Fasting glucose (mg/dL)	114.3 ± 52.6	108.1 ± 48.0	0.300 *
Hb (g/dL)	10.3 ± 1.4	10.5 ± 1.3	0.150 *
Platelet (×1000/μL)	179.4 ± 54.0	201.8 ± 58.1	0.003 *
Fe (ug/dL)	76.9 ± 37.6	75.1 ± 29.4	0.890 *
TIBC (ug/dL)	245.9 ± 45.9	236.0 ± 45.1	0.090 *
Ferritin (ng/mL)	535.3 ± 320.4	573.1 ± 252.5	0.150 *
Transferrin saturation (%)	31.5 ± 13.9	32.2 ± 12.3	0.410 *
Al (ng/mL)	6.4 ± 3.1	7.0 ± 4.4	0.460 *
Uric acid (mg/dL)	6.1 ± 1.5	6.3 ± 1.6	0.210 *
Na (meq/L)	138.1 ± 2.9	137.9 ± 3.0	0.690 *
K (meq/L)	4.6 ± 0.6	4.7 ± 0.7	0.370 *
iCa (mg/dL)	4.5 ± 0.5	4.6 ± 0.5	0.360 *
P (mg/dL)	5.1 ± 1.3	5.1 ± 1.3	0.670 *
Kt/V (Gotch)	1.4 ± 0.2	1.4 ± 0.2	0.100 *
PTH (pg/mL)	327.6 ± 306.9	245.0 ± 250.9	0.010 *
HD parameters			
SBP	147.89 ± 23.96	140.52 ± 25.56	0.025 **
DBP	70.28 ± 13.99	66.30 ± 14.53	0.035 **
MAP	96.13 ± 15.34	91.02 ± 16.45	0.016 **
Hypotension during dialysis	43 (36.8)	52 (41.9)	0.411 †
Conductivity of HD	13.99 ± 0.12	13.95 ± 0.39	0.697 *
Treatment time of HD			<0.001 ‡
4 h	103 (88)	85 (68.5)	
3.5–4 h	3 (2.6)	3 (2.4)	
3.0–3.5 h	11 (9.4)	36 (29)	
Treatment frequency			0.916 ‡
TIW	103 (88)	111 (89.5)	
BIW	13 (11.1)	12 (9.7)	
QW	1 (0.9)	1 (0.8)	
Interdialytic weight gain (kg)	2.57 ± 1.23	2.45 ± 1.05	0.656 *
Ultrafiltration (L)	2.53 ± 1.13	2.41 ± 0.97	0.630 *
Medications			
Anti-HTN drugs			
ACEI/ARB	64 (54.7)	66 (53.2)	0.818 †
β-blocker	62 (53.0)	63 (50.8)	0.734 †
Calcium channel antagonist	68 (58.1)	76 (61.3)	0.616 †
Anti-diabetic agents			
OAD (%)	36 (30.8)	35 (28.2)	0.665 †
Insulin and analogues (%)	29 (24.8)	12 (9.7)	0.002 †
Antiplatelets (%)	61 (52.1)	41 (33.1)	0.003 †
Anticoagulants (%)	6 (5.1)	4 (3.2)	0.459 ‡

PAD, peripheral artery disease; COPD, chronic obstructive pulmonary disease; AST, aspartate aminotransferase; TIBC, transferrin iron-binding capacity; PTH, parathyroid hormone; TIW, three times weekly; BIW, twice weekly; QW, once weekly; SBP, systolic blood pressure; DBP, diastolic blood pressure; MAP, mean arterial pressure; ACEI/ARB, angiotensin-converting enzyme inhibitors/angiotensin receptor blockers; OAD, oral antidiabetic drugs; Data are expressed as n (%) for categorical data and as mean ± standard deviation for continuous data. ** Student *t*-test, * Kruskal–Wallis test, † chi-square test, ‡ Fisher’s exact test.

**Table 2:** Echocardiographic features of chronic hemodialysis (HD) patients with functional arteriovenous access (AVA) between high- and low-IVCD groups. IVCD, inferior vena cava diameter.

Variables	High IVCD (*n* = 117)	Low IVCD (*n* = 124)	*p* Value
Aortic root (mm)	32.00 ± 4.33	32.18 ± 4.70	0.900 *
IVS (mm)	12.91 ± 5.01	11.37 ± 2.71	<0.001 *
LA diameter (mm)	45.08 ± 8.55	40.50 ± 7.00	<0.001 *
LVEDD (mm)	51.11 ± 7.59	47.84 ± 6.86	<0.001 *
LVESD (mm)	32.95 ± 9.12	28.38 ± 6.63	<0.001 *
LVPW (mm)	11.58 ± 3.15	10.51 ± 2.34	<0.001 *
LV mass (g)	268.02 ± 202.51	201.10 ± 79.23	<0.001 *
LVMI	162.65 ± 129.27	125.75 ± 45.28	<0.001 *
RWT (mm)	0.47 ± 0.17	0.44 ± 0.13	0.476 *

IVS, interventricular septum; LA, left atrium; LV, LVEDD, left ventricular end-diastolic diameter; LVESD, left ventricular end-systolic diameter; LVPW, left ventricular posterior wall; LVMI, left ventricular mass index; RWT, relative wall thickness; * Kruskal–Wallis test.

**Table 3:** Outcomes of chronic HD patients with functional AVA between high- and low-IVCD groups.

Inferior Vena Cava Diameter (IVCD)	Mortality	CV Mortality	MACE
Low	11.0 (8.9)	8.0 (6.5)	21.0 (16.9)
High	26.0 (22.2)	20.0 (17.1)	50.0 (42.7)
*p* Value	0.004 *	0.010 *	<0.001 *

CV, cardiovascular; MACE, major adverse cardiovascular events. Data are expressed as n (%) for categorical data. * Chi-square test.

**Table 4:** Cox regression analysis for inferior vena cava diameter (IVCD) with mortality, CV mortality, and MACE. MACE, major adverse cardiovascular events.

Event Outcome (Relative to Low IVCD)	Crude	Model 1	Model 2
	HR (95% CI)	aHR (95% CI)	aHR (95% CI)
All-cause mortality			
high IVCD	2.66 (1.31, 5.38) **	2.72 (1.34, 5.54) **	2.88 (1.06, 7.86) *
CV mortality			
high IVCD	2.81 (1.24, 6.39) *	2.69 (1.21, 5.99) **	2.85 (0.97, 8.34)
MACE			
high IVCD	2.95 (1.77, 4.92) ***	3.16 (1.84, 5.43) ***	3.42 (1.73, 6.77) ***

CV, cardiovascular; MACE, major adverse cardiovascular events; HR, hazard ratio. Model 1: adjusted for age and sex; model 2: adjusted for age, sex, coronary artery disease, total protein, alkaline-P, cholesterol, PTH, MAP, treatment time, insulin, and analogues, and antiplatelets. * *p* < 0.050, ** *p* < 0.010, *** *p* < 0.001.

**Table 5:** Subgroup Cox regression analysis for IVCD between high- and low-LVESD with all-cause mortality, CV mortality, and MACE.

Endpoints		N	Event	%	HR	(95% CI)	Interaction *p* Value
**All-cause mortality**		241	37	15.4			
**IVCD**							
High		117	26	22.2	2.66	(1.31–5.38)	
Low		124	11	8.9	1.00	(ref.)	
**IVCD**	**LVESD**						0.0575
High	High	67	14	20.9	4.60	(1.04–20.4)	
Low	High	41	2	4.9	1.00	(ref.)	
High	Low	49	12	24.5	2.50	(1.03–6.09)	
Low	Low	76	8	10.5	1.00	(ref.)	
**CV mortality**		241	28	11.6			
**IVCD**							
High		117	20	17.1	2.81	(1.24–6.39)	
Low		124	8	6.5	1.00	(ref.)	
**IVCD**	**LVESD**						0.0318
High	High	67	11	16.4	7.21	(0.93–55.6)	
Low	High	41	1	2.4	1.00	(ref.)	
High	Low	49	9	18.4	2.52	(0.90–7.03)	
Low	Low	76	6	7.9	1.00	(ref.)	
**MACE**		241	71	29.5			
**IVCD**							
High		117	50	42.7	2.95	(1.77–4.92)	
Low		124	21	16.9	1.00	(ref.)	
**IVCD**	**LVESD**						0.0042
High	High	67	31	46.3	3.88	(1.63–9.28)	
Low	High	41	6	14.6	1.00	(ref.)	
High	Low	49	19	38.8	2.34	(1.18–4.66)	
Low	Low	76	14	18.4	1.00	(ref.)	

CI, confidence interval; CV, cardiovascular; HR, hazard ratio; IVCD, inferior vena cava diameter; LVESD, left ventricular end-systolic diameter; MACE, major adverse cardiovascular events.

## Data Availability

All data generated or analyzed during this study are included in this article. Further enquiries can be directed to the corresponding author.
